# Relationship with Parents, Emotion Regulation, and Callous-Unemotional Traits in Adolescents' Internet Addiction

**DOI:** 10.1155/2018/7914261

**Published:** 2018-05-23

**Authors:** Carmen Trumello, Alessandra Babore, Carla Candelori, Mara Morelli, Dora Bianchi

**Affiliations:** ^1^Department of Psychological, Humanistic and Territorial Sciences, University “G. d'Annunzio”, Chieti, Italy; ^2^Department of Human and Social Sciences, Université de la Vallée d'Aoste, Italy; ^3^Department of Developmental and Social Psychology, Sapienza University of Rome, Italy

## Abstract

The aim of this study was to investigate the associations of relationship with parents, emotion regulation, and callous-unemotional traits with Internet addiction in a community sample of adolescents. Self-report measures of relationship with parents (both mothers and fathers), emotion regulation (in its two dimensions: cognitive reappraisal and expressive suppression), callous- unemotional traits (in its three dimensions: callousness, uncaring, and unemotional), and Internet addiction were completed by 743 adolescents aged 10 to 21 years. Results showed that a low perceived maternal availability, high cognitive reappraisal, and high callousness appeared to be predictors of Internet addiction. The implications of these findings are then discussed.

## 1. Introduction

Adolescence is known to be a period of major transformations in psychological and bodily aspects, openness to new experiences, and construction of a personal identity. In this process, an important role is played by new technologies, which attract much interest among young people, as a means to promote social relationships [1] and to freely explore a “wider world”.

The Internet, accessible through a large number of devices, is highly integrated in the daily experiences of adolescents [2, 3]. Recent studies found associations between new technology use (e.g., the Internet and video games) and typical adolescent needs, such as self-affirmation, belonging, and identity exploration [4, 5]. Despite the normative nature of and subsequent convenience provided by new technology use among adolescents, an excessive use can potentially become problematic, as it may result in serious psychosocial dysfunction, evident in arguments with and lying to relatives and friends, poor achievement, and social withdrawal [6, 7]. However, previous results on the association between time spent online and problematic Internet use, as synthesised in a meta-analysis by Tokunaga and Rains [8], are not so obvious as it might be supposed. In fact, some authors (e.g., [9]) underlined a positive relation between time using Internet and Internet addiction, while others (e.g., [10]) found only a weak association.

More generally, many authors have recently underlined the risk factors related to a problematic Internet use among adolescents [11–14], including the possibility of Internet addiction [15]. Despite the controversies about a consistent nomenclature (e.g., Internet dependency, problematic use of the Internet) and the criteria for Internet addiction, the consensus among researchers is that adolescents may especially be at risk for the detrimental use of the Internet [16] because of their limited capacity of self-regulation and their vulnerability to peer pressure [17].

The problematic use of the Internet is characterised by some aspects that are also present in other addictive disorders [18]. Block [19] particularly identified the four following components: excessive use (with a loss of one's sense of time), withdrawal (including a sense of anger and/or depression and anxiety when the Internet is not available), obsession (constant need for better digital technologies or more hours of use), and negative consequences (arguments, lying, poor achievement, and social isolation).

Consistent with this view, we use the term “Internet addiction” in this study to refer to the damaging use of the Internet as linked to dysfunctional effects on the emotive and social areas of adolescents' life [20]. As Tokunaga [21] suggested in a recent meta-analysis, clinicians described this addiction as a loss of conscious self-control over Internet use. Many studies underlined the loss of self-control in populations susceptible to addiction (e.g., anxious/depressed people), in regular Internet users [22] and, in an increasingly worrying way, among adolescents [15, 23, 24].

Research has demonstrated that difficulties in emotion regulation play an important role in situations of addictive behaviours [25, 26], problematic use of the Internet [27, 28], and pathological use of social networking sites [29]. According to Gross et al. [30], emotion regulation is a subtype of affect regulation (the other subtypes are coping and mood regulation) that refers to attempts to influence emotional experience, whether consciously or unconsciously, and may involve the up- or downregulation (i.e., increase or decrease) of various aspects of positive or negative emotions [31]. Two commonly used strategies for downregulating emotion are* cognitive reappraisal* and* expressive suppression*. Reappraisal involves changes in the way a situation is construed to decrease its emotional impact in negative emotion contexts. By contrast, suppression inhibits the external signs of inner feelings and the emotion-expressive behaviour [32].

Compared with research on adults, fewer studies have explored emotional regulation as linked to Internet addiction in adolescents, focusing mainly on the connection between emotional regulation and a problematic Internet use.

For example, in a study that involved 525 high school students, Yu et al. [33] observed that adolescents' difficulties in emotion regulation were significantly associated with problematic Internet use. Another recent research [34], conducted on a sample of 380 adolescent students from a secondary school in Italy, confirmed that emotion regulation was negatively correlated with a dysfunctional use of the Internet. However, there is a lack of knowledge regarding which emotion regulation strategy plays a major role in these dysfunctional behaviours.

Another important aspect studied in empirical research on adolescents' Internet addiction is the possible influence of relationship with parents. In the literature, the consensus is that adolescents' relationship with their parents may deeply influence their psychosocial well-being [35, 36] and life satisfaction and that different parenting styles are linked to different psychosocial profiles, particularly in the self-concept domain [37].

In several studies, Yu and Gamble [38–40] stated that adolescents who perceived their parents as warmer and nonrejecting were less likely to exhibit negative or problematic psychosocial behaviours. On the contrary, adolescents whose parents were perceived to be more rejecting and unsupportive demonstrated a greater risk for problematic behaviours and psychosocial outcomes, such as Internet addiction [41].

Several studies have investigated the correlation between the quality of parent–adolescent relationship and problematic Internet use. For example, positive parental relationships were found to be negatively correlated with Internet addiction [42], whereas conflictual relationships were positively associated with Internet addiction [43]. In the presence of Internet addiction, adolescents described their parents as lacking in warmth, more intrusive, rejecting, and punitive [44].

However, while numerous studies have examined the connection between Internet addiction in adolescence and quality of parental relationship, research with a specific focus on adolescents' Internet addiction and parents' perceived emotional availability [45] is lacking.

Emotional availability refers to a set of aspects (e.g., support, sensitivity, and responsiveness) that relate to the emotional bond with parents [46]. According to Lum and Phares [45], this construct is consistent with theoretical and empirical evidence that emotionally available parents are related to a child's greater sense of security, promoting the development of personal and interpersonal competencies.

All these aspects suggest the importance of exploring different factors, individual and interpersonal, which may increase or decrease the risk of adolescents' problematic Internet use.

In this frame, we examined an additional factor, the role of callous-unemotional (CU) traits, which, in recent years, have been the focus of much attention in studies on behavioural problems [47, 48]. CU traits can be defined as a set of characteristics including lack of empathy, remorse and guilt, and lack of concern about the negative impacts of one's own actions [49]. The growing interest on CU trait assessment resulted in the development of the Inventory of Callous-Unemotional Traits (ICU) by Frick and colleagues [50, 51]. The three following dimensions are analysed with the use of this widely used self-report tool:* callousness*, which refers to the lack of empathy, guilt, and remorse for misdeeds;* uncaring*, which encompasses lack of care for one's own actions and for other people's feelings; and* unemotional*, which refers to shallow or deficient affects [52, 53].

Although CU traits have been traditionally considered the core affective dimension of psychopathy, several studies found their association with poor peer relationships, low prosociality, and psychosocial maladjustment in community adolescents [47, 53, 54]. Previous research also highlighted the relation of CU traits with aggressiveness, drug consumption, and multiple psychosocial impairments in children and adolescents [48, 55]. Commenting on the findings of studies on this topic, Viding and McCrory [54] suggested that CU traits may, overall, be considered a risk factor for poorer social functioning.

So far, despite previous research showing the association of CU traits with psychosocial maladjustment, no published study has focused on the relationship between CU traits and Internet addiction.

Starting from these premises, the current research pursued different goals. First, we aimed to analyse the associations between the use of new technologies and the Internet addiction. Given the results of existing research, we expect a positive correlation between these two variables.

Second, we aimed to explore if and to what extent three specific areas, namely, perceptions of parenting relationship (in terms of emotional availability), emotion regulation strategies (cognitive reappraisal and expressive suppression), and CU traits (callousness, unemotional, and uncaring), may be related to Internet addiction. Specifically, we hypothesised that perceptions of a low parenting relationship and high levels of CU traits could be possible predictors of Internet addiction.

With regard to emotion regulation, even if other studies have explored this issue, the particular emotion regulation strategy that may be involved in Internet addiction during adolescence is unclear. Thus, in this study, we aimed not only to explore the relation between emotional regulation and Internet addiction in this particular phase of development but also to verify whether a particular emotion regulation strategy (cognitive reappraisal and/or expressive suppression) may be predictive of Internet addiction. Given the lack of specific research exploring individual emotion regulation strategies, we do not advance any hypothesis on whether and what particular emotion regulation strategy may have a predictive power for Internet addiction.

## 2. Materials and Methods

### 2.1. Participants

The study was conducted on 743 Italian participants aged 10 to 21 years (M_age_ = 15.64; SD_age_ = 2.08; 57.6% females). They were recruited in public schools in urban and suburban areas of Chieti and Rome. Paper-and-pencil questionnaires were administered, and all participants gave their informed consent in accordance with the Declaration of Helsinki. For underage participants, written informed consent was also obtained from their parents and from school authorities. The Ethics Committee of the Department of Psychological Sciences of the University approved this study.

### 2.2. Measures

*Sociodemographic Data*. The participants reported their age, gender, and information regarding their family.

*New Technology Use*. Nine items selected from the Questionnaire about New Digital Technologies (QNDT) were administered to evaluate some behaviours related to Internet and video game use. Two items measured the amount of time spent online daily and the amount of time spent on video games daily on a four-point Likert scale ranging from 1* (less than 1 hour)* to 4* (more than 3 hours)*, and five items assessed the role of parents in Internet use on a four-point Likert scale ranging from 1* (never)* to 4* (very often)*. Specifically, the items investigated playing video games with the mother, playing video games with the father, using the Internet with the mother, using the Internet with the father, and quarrels with parents on the amount of time spent on the Internet or on video games. Finally, two dichotomous items evaluated parental restriction and making friends online (0 =* no *and 1 =* yes*).

*Perceived Emotional Availability of Parents*. To evaluate the participants' perception of maternal and paternal emotional availability, we administered the two versions of the Lum Emotional Availability of Parents (LEAP) questionnaire [45, 56, 57]. Each version (LEAP-mother and LEAP-father) comprised the same 15 items rated on a six-point Likert scale ranging from 1* (never)* to 6* (always)*. A higher score reflects a higher level of perceived emotional availability. Both LEAP-mother and LEAP-father reached good reliability, with a Cronbach's alpha of .94 and .96, respectively. 

*Emotion Regulation*. The Emotion Regulation Questionnaire (ERQ) [58, 59] was administered to assess expressive suppression and cognitive reappraisal, two specific emotion regulation strategies which are typically used to manage positive and negative emotions in daily life. The expressive suppression scale is composed of four items evaluating the inhibition of emotion-expressive behaviour. The cognitive reappraisal scale is composed of six items evaluating the cognitive ability of modifying the meaning and the emotional impact of a situation. All items were rated on a seven-point Likert scale ranging from 1* (strongly disagree)* to 7* (strongly agree)*. The expressive suppression scale and the cognitive reappraisal scale obtained a Cronbach's alpha of .60 and .79, respectively. 

*Callous-Unemotional Traits*. The Inventory of Callous-Unemotional Traits (ICU) [50, 52] is a self-report questionnaire that evaluates callous-unemotional traits, which were theorised by Frick et al. [60]. These traits are the affective dimension of psychopathy [61] and refer to the lack of empathy, guilt, and any emotional expression. The factor structure of the scale showed the presence of three subscales: callousness (nine items), unemotional (five items), and uncaring (eight items).

“Callousness” assessed the lack of empathy and remorse (Cronbach's alpha of .68), “unemotional” assessed the lack of emotional expression (Cronbach's alpha of .59), and “uncaring” assessed the insensibility towards others' emotions and task performance (Cronbach's alpha of .70). All items were rated on a four-point Likert scale ranging from 0* (not at all true)* to 3* (definitely true)*.

*Internet Addiction*. Internet addiction was evaluated with the 10-item Internet addiction subscale added to the Shorter Promis Questionnaire (SPQ) [62] in the SPQ Italian validation [63]. This scale measured several indexes of Internet addiction, such as difficulty in stopping Internet use, concerns of others about Internet use, and the feeling of safety and emotional relief provided by Internet use. In this study, we used only nine items; we removed the item “I connect to the Internet several times during the day” because more than 75% of the participants answered above 3 on a six-point Likert scale ranging from 1* (not at all true)* to 6* (definitely true)*. This psychometric problem could be due to the current lack of discriminant validity of this item. Nowadays, people are continuously connected to the Internet through their smartphone. Thus, the final scale, composed of nine items, reached a good reliability, with a Cronbach's alpha of .74.

### 2.3. Data Analysis

For descriptive purposes, we computed the correlations among the nine variables of the QNDT and Internet addiction to explore how the use of the Internet and video games and the role of parents are related to Internet addiction among young people. Then, the correlations among perceived maternal and paternal availability (LEAP-mother and LEAP-father), the two ERQ dimensions (cognitive reappraisal and expressive suppression), the three ICU dimensions (callousness, unemotional, and uncaring), and Internet addiction were computed. Afterwards, a multiple linear regression analysis was run to investigate whether perceived maternal and paternal availability, emotion regulation strategies, and callous-unemotional traits could lead to Internet addiction. In our study, we considered all investigated variables (perceived maternal and paternal availability, two emotion regulation strategies, and three callous-unemotional traits) as predictors, according to J. Cohen and P. Cohen [64] and Cohen et al. [65]. In fact, according to them, in regression analysis, the criteria (dependent variable) are regressed on the predictors (independent variables), and these terms avoid the possible interpretation of casual effects among variables.

## 3. Results

### 3.1. Correlations between Internet Addiction and New Technology Use

Internet addiction was negatively and weakly related to age. Conversely, Internet addiction was positively and modestly related to the amount of time spent online daily, the amount of time spent on video games daily, making friends online, and quarrels with parents on the amount of time spent on the Internet or on video games. On the contrary, Internet addiction was not related to gender, playing video games with the mother, playing video games with the father, using the Internet with the mother, using the Internet with the father, and parental restriction. The correlations and descriptive statistics are reported in Table 1.

### 3.2. Correlations among Perceived Parental Availability, Emotion Regulation Strategies, Callous-Unemotional Traits, and Internet Addiction

Internet addiction was negatively and weakly related to perceived maternal availability. Conversely, Internet addiction was positively and weakly related to cognitive reappraisal and expressive suppression. Moreover, Internet addiction was positively and modestly related to callousness. The correlations and descriptive statistics are reported in Table 2.

### 3.3. Multiple Linear Regression Analysis

A multiple linear regression analysis was run to determine whether perceived maternal and paternal availability, emotion regulation strategies (i.e., cognitive reappraisal and expressive suppression), and callous-unemotional traits (i.e., callousness, unemotional, and uncaring) could lead to Internet addiction. Perceived maternal availability,* beta *= −.10, *p* = .02; cognitive reappraisal,* beta *= .08,* p *= .03; and callousness,* beta *= .24, *p* < .001 turned out to be significant predictors of Internet addiction, accounting for 8.1% of the variance,* R *= .28, *p* < .001. Thus, low maternal availability, high cognitive reappraisal, and high callousness appeared to be predictors of Internet addiction. The full statistics of the model are reported in Table 3.

## 4. Discussion

This study was developed to explore the association of Internet addiction with the use of new technologies, relationship with parents (both mothers and fathers), emotion regulation (in its two dimensions: cognitive reappraisal and expressive suppression), and callous-unemotional traits (in its three dimensions: callousness, uncaring, and unemotional) among Italian adolescents.

For the first aim, our data showed a positive but modest relationship of time spent on video games and online with Internet addiction. Existing research on this topic produced mixed results [9, 10, 66]. In his analysis Block [19] suggested that an excessive time spent on the Internet characterises Internet addiction, while following Gamito et al.'s suggestion [67], a simple frequency of use could not be an indicator of addiction or risk of addiction, since specific uses of the Internet may be addictive. Hence, it might not be appropriate to refer only to the amount of time spent online as the criterion for identifying Internet addiction, because adolescents may use the Internet for different purposes [10], spending a lot of time online, without missing their ability to control this activity.

Therefore, in order to distinguish between adaptive and maladaptive use of the Internet, other processes should be explored, such as emotional and self-regulatory ones.

With regard to emotion regulation, we found a predictive association between cognitive reappraisal and Internet addiction. According to Gross' process model of emotion regulation [68] and his studies on the affective, cognitive, and social consequences of emotion regulation strategies [32], reappraisal is associated with a greater positive emotion experience and expression and a lesser negative emotion experience than suppression is. Therefore, as cognitive reappraisal involves reinterpreting negative emotional stimuli in a nonemotional manner [68], Internet overuse seems to be an attempt of adolescents to avoid difficult emotions through instant emotional gratification or avoidance, distraction, and disengagement [26, 69]. In fact, as Spada et al. [70] stated, an excessive use of the Internet may be a form of maladaptive self-regulatory strategy because it may be useful for distracting adolescents from negative affective states. Besides, contrary to suppression, cognitive reappraisal is evoked early in the emotion generative process, and it is not necessarily a continual self-regulatory effort during an emotional event [32]. As adolescents experience continuous emotional changes and the need for instant emotional gratification, reappraisal can be hypothesised as the most immediate strategy that can be used towards negative emotions.

Our results provide an important contribution to the literature on Internet addiction in adolescents because they showed the predictive power of a specific emotion regulation strategy (cognitive reappraisal) in Internet addiction. However, our findings must be considered with caution, and they require further study.

Indeed, adolescence is a period of emotive and psychosocial changes, characterised by a hyperactivated emotional–behavioural functioning with a major tendency towards action [71], especially if one lacks self-regulatory strategies [2]. In this phase, because of the lesser emotion self-control capacity, adolescents may be more vulnerable to and at risk for developing addictive behaviours, such as Internet addiction, and other problematic behaviours [72–74].

According to our findings, the callousness dimension was the strongest predictor of Internet addiction among the variables we considered. The two other dimensions encompassed in the ICU, unemotional and uncaring, were not significantly related to Internet addiction. This result is quite new because, to our knowledge, this is the first study to analyse the role of CU traits in this kind of addiction. Among other forms of addictive behaviours, CU traits were found in a prospective study by Wymbs et al. [75] to predict substance use among early adolescent boys; more recently, Romero and Alonso [48] found that, compared with the uncaring and unemotional dimensions, callousness was the strongest predictor of alcohol and cannabis consumption among a sample of Spanish adolescents. The callousness dimension was also associated with gambling disorder in a sample of nonreferred Italian gamblers [76]. These last two studies emphasised the importance of callousness in the comprehension of addictive disorders, a result that resembles that of the current study. More specifically, Internet addiction is often characterised by social isolation and withdrawal, a feature that might be consistent with our findings of a positive correlation with callousness. In fact, callousness may be considered as the CU trait dimension most closely related to lack of empathy [48].

Even if this did not fall within the objectives of the study, our data showed a highly positive association between the unemotional dimension of CU traits and the expressive suppression strategy of emotional regulation. This finding highlights an overlap between these two constructs, which is not surprising because adolescents with CU traits are classically defined as lacking emotional expression [61] and having limited emotional depth [77] and deficits in emotional processing [78]. Furthermore, we analysed the connection between Internet addiction and the emotional quality of relationship with parents, both mothers and fathers. Our results showed that maternal emotional availability, but not paternal emotional availability, predicted Internet addiction. More specifically, lower levels of emotional quality in maternal relationship were associated with higher levels of Internet addiction. This result is quite new, as previous research did not specifically consider the construct of parental emotional availability and instead focused on the more general parent–child relationship quality. A recent review by Schneider et al. [79] analysed 11 previous studies showing that a poorer quality of parent–child bond correlated with an increased severity in problematic Internet gaming. Moreover, previous studies focusing on adolescents' overall psychological adjustment highlighted that maternal parenting played a more relevant role than paternal parenting did [80, 81]. Overall, an inadequate maternal emotional availability and unemotional exchanges between mother and child may be argued to lead to negative relationships with others, self-perceived loneliness, and sadness [82], which, in turn, may predict the development of Internet addiction [2].

Some limitations of this study have to be considered. First, the exclusive use of self-report tools and the absence of other informants (e.g., parents and/or teachers) do not address the issue of shared method variance bias. Moreover, because of the cross-sectional nature of the research, no causal relationships among the considered variables may be inferred. Lastly, as our data refer to an Italian sample of adolescents, the generalisation of the findings to other countries cannot be done.

Nevertheless, the present research made a relevant contribution to knowledge on the significant factors associated with Internet addiction. In particular, our study was the first to analyse the role played by parental emotional availability, specific emotion regulation strategies, and callous-unemotional traits in Internet addiction.

## 5. Conclusion

Nowadays, Internet addiction is increasingly studied because of the negative effects this addiction may have on adolescents; it results in a higher risk for aggressive behaviours [83], poorer school performance [84], low quality of interpersonal relationships [85, 86], and an increase in depressive symptoms [15, 87]. Furthermore, adolescents with Internet addiction are more exposed to other important risks linked to the world of the Web, such as cyber bullying, sexual solicitation, and identity theft.

The early recognition of Internet addiction among adolescents and the possibility of preventing this addictive behaviour are crucial. As Di Nicola et al. [88] underlined in a recent study, the last European School Survey Project on Alcohol and Other Drugs [89] report of 2016 stated that “since the Internet has become an integral part of life and is used on a daily basis, the development of patterns of addictive use among children and adolescents needs to be closely monitored and investigated in further studies” and that “measures to prevent adolescents from developing problems associated with gambling, such as debts, psychological deficits and social disadvantages, are of high priority”.

## Figures and Tables

**Table 1:** Correlations among Internet addiction and new technology use.

	1	2	3	4	5	6	7	8	9	10	11	12	M	SD
(1) Gender	1													
(2) Age	.10^*∗∗*^	1											15.64	2.08
(3) Time online_QNDT	.16^*∗∗∗*^	.21^*∗∗∗*^	1										2.85	1.03
(4) Time on videogames_QNDT	−.46^*∗∗∗*^	−.24^*∗∗∗*^	−.02	1									1.49	0.81
(5) Videogames with mother_QNDT	.17^*∗∗∗*^	−.08^*∗*^	.02	−.001	1								1.42	0.62
(6) Videogames with father_QNDT	−.10^*∗*^	−.20^*∗∗∗*^	−.08^*∗*^	.18^*∗∗∗*^	.43^*∗∗∗*^	1							1.63	0.72
(7) Internet with mother_QNDT	.15^*∗∗∗*^	.03	.07	−.12^*∗∗*^	.34^*∗∗∗*^	.15^*∗∗∗*^	1						2.18	0.75
(8) Internet with father_QNDT	−.10^*∗∗*^	−.03	−.001	.07^*∗*^	.19^*∗∗∗*^	.33^*∗∗∗*^	.47^*∗∗∗*^	1					2.10	0.79
(9) Quarrels with parents_QNDT	.01	−.02	.26^*∗∗∗*^	.06	−.05	−.04	−.03	−.04	1				1.74	0.86
(10) Parental restriction_QNDT	−.11^*∗∗*^	−.20^*∗∗∗*^	−.06	.07^*∗*^	.003	.001	−.03	.01	.05	1			0.46	0.51
(11) Making friends online_QNDT	.07	.14^*∗∗∗*^	.22^*∗∗∗*^	.06	−.04	−.02	−.06	−.01	.10^*∗*^	−.01	1		0.61	0.49
(12) Internet addiction_SPQ	−.03	−.10^*∗∗*^	.25^*∗∗∗*^	.19^*∗∗∗*^	−.01	.01	−.05	−.01	.25^*∗∗∗*^	.02	.15^*∗∗∗*^	1	20.50	7.49

*Note*. ^*∗∗∗*^*p* < .001; ^*∗∗*^*p* < .01; ^*∗*^*p* < .05. Gender was coded as 0 = males and 1 = females.

**Table 2:** Correlations among perceived parental availability, ICU dimensions, ERQ dimensions, and Internet addiction.

	1	2	3	4	5	6	7	8	M	SD
(1) LEAP-mother	1								71.91	13.94
(2) LEAP-father	.47^*∗∗∗*^	1							64.47	17.78
(3) Callousness_ICU	−.11^*∗∗*^	−.03	1						5.36	4.14
(4) Unemotional_ICU	−.14^*∗∗∗*^	−.11^*∗∗*^	.19^*∗∗∗*^	1					7.31	2.97
(5) Uncaring_ICU	−.26^*∗∗∗*^	−.16^*∗∗∗*^	.34^*∗∗∗*^	.24^*∗∗∗*^	1				7.59	3.94
(6) Cognitive reappraisal_ERQ	.12^*∗∗*^	.15^*∗∗∗*^	−.01	−.03	−.24^*∗∗∗*^	1			26.55	7.16
(7) Expressive suppression_ERQ	−.07	−.02	.18^*∗∗∗*^	.49^*∗∗∗*^	.03	.18^*∗∗∗*^	1		14.90	5.00
(8) Internet addiction_SPQ	−.11^*∗∗*^	−.05	.24^*∗∗∗*^	.03	.04	.09^*∗*^	.10^*∗∗*^	1	20.50	7.49

*Note*. ^*∗∗∗*^*p* < .001; ^*∗∗*^*p* < .01; ^*∗*^*p* < .05.

**Table 3:** Multiple linear regression analysis (*N* = 743).

Internet addiction		
Predictor		Beta
LEAP-mother		−.10^*∗*^
LEAP-father		−.02
Callousness		.24^*∗∗*^
Unemotional		−.05
Uncaring		−.04
Cognitive reappraisal		.08^*∗*^
Expressive suppression		.07
Total *R*^2^	.08^*∗∗*^	

*Note*. ^*∗*^*p* < .05; ^*∗∗*^*p* < .001. The interaction effects between all investigated variables (perceived maternal and paternal availability, the two emotion regulation strategies, and the three callous-unemotional traits) and gender, and between all investigated variables (perceived maternal and paternal availability, the two emotion regulation strategies, and the three callous-unemotional traits) and age were tested to examine the possible moderating effects of gender and age. An initial hierarchical regression analysis was run to verify the interaction effects between gender and all investigated variables on Internet addiction. The interaction terms were entered in the second step, but no significant interaction effects were found. Another hierarchical regression analysis was run to verify the interaction effects between age and all investigated variables on Internet addiction. The interaction terms were also entered in the second step, but no significant interaction effects were found.
